# Cardiac myosin inhibitors in hypertrophic cardiomyopathy: advancing care with caution

**DOI:** 10.1097/MS9.0000000000003981

**Published:** 2025-10-06

**Authors:** Zoya Rehman, Faisal Ahsaan, Bakhtawar Latif, Ahmad Furqan Anjum, Hermann Yokolo

**Affiliations:** a Department of Internal Medicine, Shaikh Zayed Postgraduate Medical Institute (SZPGMI), Lahore, Pakistan; b Department of Research, Medical Research Circle (MedReC), Goma, DR Congo.

*To the Editor,*

We read with great interest the systematic review and meta-analysis by Hasana *et al*^[1]^, which offers valuable insights into the clinical use of cardiac myosin inhibitors (CMIs) in hypertrophic cardiomyopathy (HCM). The authors effectively synthesize randomized controlled trial (RCT) data to support CMIs as a new treatment class targeting the main issues in HCM, namely sarcomeric hypercontractility. Although this systematic review makes an important contribution by highlighting the potential of CMIs in HCM, gray areas remain regarding their safety, cost-effectiveness, and place relative to standard treatments. The objective of our article is to provide critical insight into these limitations, to emphasize the need for data from diverse populations, and to emphasize the importance of comparative studies and rigorous follow-up to optimize the integration of MICs into clinical practice. This article complies with the TITAN 2025 guidelines – repressing the reporting and use of AI^[2]^.

However, several limitations deserve further discussion. First, the safety profile of CMIs needs closer examination. While agents like mavacamten and aficamten lower left ventricular outflow tract gradients and enhance functional capacity, the risk of systolic dysfunction is significant. In the EXPLORER-HCM trial, 7.5% of patients taking mavacamten experienced a left ventricular ejection fraction (LVEF) below 50%, necessitating temporary discontinuation of treatment^[3]^. Recent follow-up data indicate that reversible LVEF drops can occur even with aficamten, despite its shorter half-life and more predictable pharmacokinetics^[4]^. Second, the generalizability of the included studies is very limited. Most RCTs in the review involved middle-aged White patients from wealthy countries and lacked representation of ethnically diverse populations. This raises concern because HCM phenotypes and clinical outcomes can differ significantly by race and ethnicity^[5]^. Future trials must include a wider range of geographic and demographic groups. Third, while the review emphasizes efficacy metrics, it offers little discussion on how CMIs compare to surgical or catheter-based procedures like septal myectomy or alcohol septal ablation. These traditional methods are still considered gold standards, especially for patients with severe obstructive symptoms. A 2021 study by Ommen *et al* confirmed the durability and mortality benefits of septal myectomy, particularly in younger and symptomatic patients^[6]^. Without head-to-head comparisons, the long-term role of CMIs will continue to remain unclear. Another major factor is the cost ineffectiveness of CMIs, which presents a significant barrier to their widespread use. Early economic models suggest that annual costs for mavacamten exceed $75 000 per patient, with uncertain long-term cost benefits, particularly in health systems outside North America and Europe^[7]^. These costs may restrict access in low-resource areas where surgical options will continue to remain more viable and sustainable. Lastly, the review could advocate more strongly for structured monitoring during treatment. Serial echocardiograms, electrocardiograms (ECGs), and pharmacokinetic monitoring are crucial for detecting early contractility suppression or arrhythmia risk, especially when CMIs are used alongside beta-blockers or antiarrhythmics.

In conclusion, the review by Hasana *et al* is a valuable addition to the literature and highlights the advances in HCM management. However, we stress the need for long-term outcome data, inclusive trial designs, comparative efficacy studies, particularly with gold standard treatment modalities, and a thorough cost-effectiveness analysis before CMIs can become first-line treatments in routine clinical practice.

## Data Availability

Not applicable.
